# Self-assembling peptide and nano-silver fluoride in remineralizing early enamel carious lesions: randomized controlled clinical trial

**DOI:** 10.1186/s12903-023-03269-4

**Published:** 2023-08-19

**Authors:** Sara M. Atteya, Hala A. Amer, Susan M. Saleh, Yara Safwat

**Affiliations:** 1 Department of Pediatric Dentistry and Dental Public Health, Faculty of Dentistry, Alexandria University, Champollion St., Azarita, 21527 Alexandria Egypt; 2 Department of Medical Microbiology and Immunology, Faculty of Medicine, Alexandria University, Alexandria, Egypt

**Keywords:** Self-assembling peptide, P11-4, Nano silver fluoride, Sodium fluoride, White spot lesions, ICDAS, Diagnodent

## Abstract

**Background:**

Nanoparticles and regenerative biomineralization are new caries prevention technologies. This study assessed the remineralizing effect of self-assembling peptide (P11-4), Nanosilver Fluoride (NSF) and sodium fluoride (NaF) on white spot lesions (WSLs) in permanent teeth.

**Methods:**

Sixty six young adults with WSLs on buccal surfaces in permanent teeth and ICDAS code 1 or 2, were randomly assigned to one of three groups; P11-4, NSF or NaF. Assessment of ICDAS scores, lesion activity (Nyvad scores) and diagnodent readings of lesions were done at baseline and after 1, 3, 6 and 12 months of agents’ application. Comparisons between groups were made using chi squared test and comparison within groups were made using McNemar test. Multilevel binary logistic regression was used to assess the effect of agents on change of ICDAS scores after 3, 6 and 12 months (reduction versus no reduction).

**Results:**

There were 147 teeth in 66 patients; mean ± SD age = 13.46 ± 4.31 years. There were significant differences in the change of ICDAS scores among the three groups after 3 and 6 months (*p* = 0.005). The reduction in ICDAS score increased steadily in all groups across time with the greatest increase in the P11-4 group: 54.5% after 12 months. Lesion activity (Nyvad scores) showed significant differences among the three groups with the greatest percentage of inactive cases in the P11-4 group. Multilevel binary logistic regression showed non-significant reduction of ICDAS in P11-4 and NSF varnishes compared to NaF varnish (AOR = 2.56, 95% CI: 0.58, 8.77 and AOR = 2.12, 95% CI: 0.59, 7.64 respectively).

**Conclusion:**

P11-4 and NSF varnish reduced the ICDAS scores, caries activity and diagnodent readings of WSLs in permanent teeth. However, the change in ICDAS scores was not significantly different from NaF.

**Trial registration:**

This trial was prospectively registered on the clinicaltrials.gov registry with ID: NCT04929509 on 18/6/2021.

## Introduction

Dental caries is a multifactorial chronic disease and a major public health problem [1]. Conventional methods for caries prevention, such as topical fluoride application, decrease demineralization through fluoride deposition in the enamel crystal lattice, reducing its solubility [2]. The effect of fluoride is limited to the outer -30 μm. Consequently, fluoride does not enter deeper than the subsurface demineralized zone; where remineralisation is much needed for the affected enamel [3].

Nanotechnology is an emerging trend in dentistry. Silver nanoparticles (AgNPs) are products with high surface area and a micro- size of atoms [4]. Nano-silver fluoride (NSF) varnishes have been developed with high remineralization property and antimicrobial effect [5]. Primary teeth treated with fluoride varnish with silver nano particles had better structure than those treated with conventional varnish following remineralization of early WSLs [6]. Nano-silver mouthwash has been proved to be more effective than NaF mouthwash in reducing WSLs in permanent teeth after six months [7].

Self-Assembling Peptide (P11-4) restores hydroxyapatite crystals within the subsurface carious lesion by Guided Enamel Remineralisation (GER). It has hierarchical self-assembly property and forms a fibrillar scaffold with a 3D matrix in the presence of high ionic strength and acidic pH in carious lesions [8–10]. The safety of P11-4 was demonstrated in a trial for caries arrest in adults [11]. Clinical studies of P11-4 showed mixed results. In one study, there was a significant improvement in all outcomes after 6 months of using P11-4 combined with fluoride varnish than fluoride varnish alone in early caries on erupting permanent molars [12]. Also, P11-4 with fluoride varnish significantly decreased the size of early carious lesions in young adults compared to fluoride varnish [13]. P11-4, applied either with fluoride varnish or alone, was superior in treating early occlusal WSLs in children and adults than fluoride varnish alone [14]. Two systematic reviews of seven clinical trials showed that P11-4 improved caries arrest, decreased lesion size and lowered ICDAS scores [15, 16]. However, P11-4 alone had less remineralization effect than highly concentrated sodium fluoride agent and Remin Pro forte (fluoride, hydroxyapatite and xylitol paste) on WSLs in permanent teeth [17]. P11-4 induced less lesion regression of white spot buccal lesions in permanent than fluoride varnish [18].

P11-4 and NSF are two novel caries preventive and remineralization agents for early caries lesions. They have limited evidence base to support their relative effects with scarce information from clinical trials comparing both agents directly. Such trials can provide answers to questions about which agent to use in situations where white spot lesions in permanent teeth are present. This study compared P11-4, NSF varnish and NaF varnish in remineralizing WSLs on the buccal surfaces of permanent teeth in young adults after one, three, six and twelve months follow-up. The null hypothesis was that there would be no significant differences in lesion arrest among the three agents.

## Material and methods

### Ethical consideration and study design

This study was a parallel randomized, three arm, clinical trial conducted in the Pediatric Dentistry clinic of the Faculty of Dentistry, Alexandria University. Ethical approval was obtained from the Research Ethics Committee, Faculty of Dentistry, Alexandria University (#0086–11/2019). The trial was prospectively registered on the clinicaltrials.gov registry (NCT04929509). Before the intervention, the study was explained to the participants and they were informed about the etiology of WSLs and the available options for treatment. They were also advised about the importance of oral hygiene and proper diet in preventing WSLs lesions.

### Participant eligibility

Participants were recruited randomly from the outpatients clinic of Faculty of Dentistry, Alexandria University if they were aged 10–24 years [19], with at least one visible WSL in the buccal surface of permanent teeth, with ICDAS II score of 1 or 2, and after signing an informed consent to participate in the study, themselves if they were > 18 years of age or by their guardians/parents if they were younger [18]. The exclusion criteria were patients receiving tetracyclines, any other medication known to stain teeth [18, 20] or had fluoride application less than 3 months before the study [12]. Teeth were excluded if they had microcavities or dentinal involvement due to loss of enamel, had a restoration adjacent to the lesion to avoid diagnodent false-positive readings [18], had discolouration, enamel hypoplasia or fluorosis [18].

### Sample size calculation

Sample size was estimated based on assuming 95% confidence level and 80% study power. The percentage of inactive lesions expected after 12 months of applying P11-4 and NaF varnish were estimated to be 68.8% and 25.9% respectively [18, 21]. By comparing proportions, sample size was calculated to be 20 patients per group, with each patient having at least one active lesion. This was increased to 22 patients to make up for loss to follow up with total sample of 66 patients.

### Randomization, allocation and blinding

Participants were randomly assigned with equal allocation to the three arms, Curodont Repair (P11-4), NSF varnish and NaF varnish, using a computer-generated list of random numbers in blocks of 3 [22]. Participants were given serial numbers that was written on identical sheets of paper with the group to which the patient was allocated and placed inside an opaque envelop carrying the patient’s name. A trial independent person kept the envelopes and unfolded them at the time of the intervention. The examiner administering the intervention (NN) was not blinded, since the method of application is different for the three agents. However, the participants were blinded to the agent they received by removing the labels from the bottles. The outcome assessor (SA) was blinded to the intervention type.

### Training and calibration

The examiner (S.A.) was trained for ICDAS II and Lesion activity assessment (LAA) criteria by two senior researchers (H.A. and S.S.). Intra examiner reliability was excellent for ICDAS II and LAA examinations (Kappa statistic = 0.92 and 0.90 respectively).

### Intervention

Participants in the first group received P11-4 (Curodont Repair™, Biomedical products for tooth preservation) only at baseline. First, the tooth was completely isolated using rubber dam followed by cleaning of the tooth surface with sodium hypochlorite 2% for 20 s, acid etching with phosphoric acid gel 35–37% for 20 s, rinsing and drying. The agent was applied by removing the safety clip, activating the applicator and squeezing out the sponge above the lesion and the solution was left to diffuse for 5 min [23].

In the second group, each tooth received two drops of NSF only at baseline. NSF was prepared at the Faculty of Pharmacy, Alexandria University according to Wei et al. method [24]. It was applied with a micro brush, equivalent to a dose of 10 mg and the solution was left in contact with the tooth for 2 min [25].

The third group received 5% NaF varnish (Duraflor®) at baseline and after 6 months. The teeth were dried and a very thin coat of the varnish was applied and allowed to dry for 10 s [18].

All participants were instructed to eat only soft foods and avoid hot liquids for two hours. Oral health instructions were given to all participants.

### Outcome assessment

**Fig. 1:**
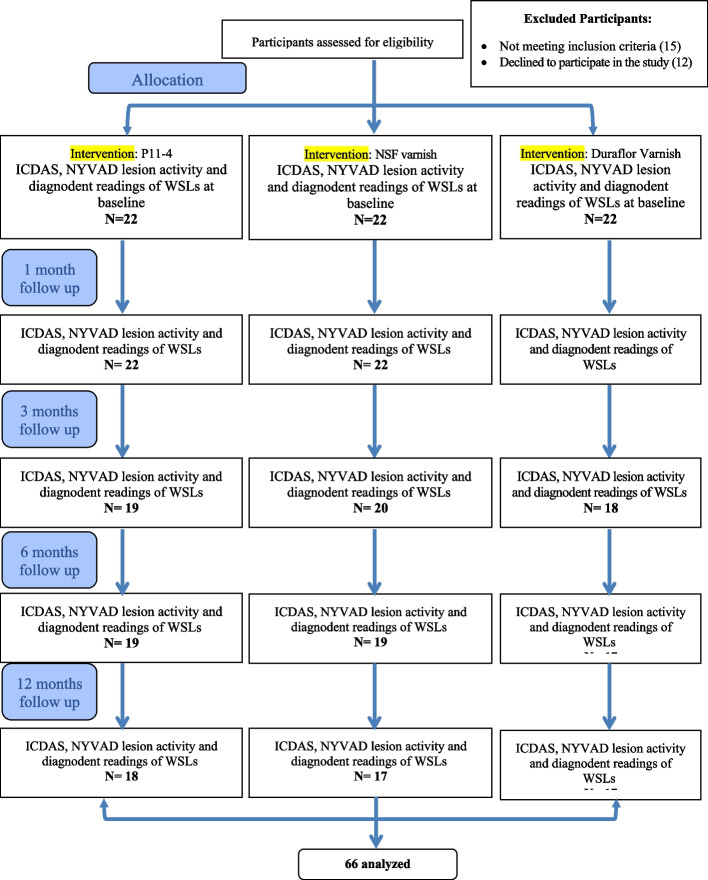
Participants flow cart

Remineralization was assessed using visual tactile assessment of WSLs based on change in ICDAS II scores (reduction from score 2 to 1, or from score 1 to 0) and LAA (NYVAD) [26] (Table 1). This was the primary outcome. DIAGNOdent score was the secondary outcome. The DIAGNOdent score ranges from 0 to 99 with higher score indicating deeper caries. A decrease in value indicates regression of the carious lesion, whereas an increase indicates progression [27]. A base line zero value for each patient is obtained by choosing a clear non carious patch of enamel, usually the middle third of an anterior tooth. The pen is pointed in perpendicular direction to this area and the set button is touched for 2 s until 0 appears, then the button is released, and zero baseline value is, thus, set. Probe B of laser fluorescence for smooth surface caries was used for lesions examination. Using cotton roll isolation and after air drying with an air syringe, the measurement was performed with the tip making contact at a right angle to the white spot lesion. Measurements were made three times and the values were averaged [28]. Outcome assessment was done at baseline and 1, 3, 6 and 12 months. The flow chart of patients and outcome assessment at each follow-up period is illustrated in Fig. 1.

**Table 1:** Lesion activity assessment (LAA) (NYVAD) criteria [27]

Clinical parameter 1 (visual appearance: severity score)	ICDAS score 1,2 (brown lesions)	1 point
	ICDAS score 1,2 (white lesions)	3 points
	ICDAS score 3, 4, 5 or 6	4 points
Clinical parameter 2 (gingival inflammation with plaque stagnation)	Gingival inflammation with plaque stagnation	3 points
	No gingival inflammation	1 point
Clinical parameter 3 (surface texture)	Rough or soft surface on gentle probing	4 points
	Smooth or hard surface on gentle probing	2 points
Final sum	≤ 7	Inactive caries
	> 7	Active caries

The World Health Organization questionnaire was used to assess participants’ oral health practices including dental visits, use of fluoridated toothpaste and frequency sugar consumption, and their socioeconomic background (age, sex) as confounders [29].

### Statistical analysis

Normality was checked using Kolmogorov–Smirnov test. Non-normally distributed variables were presented using medians and inter quartile ranges (IQR). Intention to treat analysis was used. Chi squared test was used to compare changes in ICDAS and NYVAD scores among groups and McNemar test was used to compare changes across time within the same group. For LAA, a score of more than 7 was considered active and less than 7 indicated inactive lesion [21]. Multilevel binary logistic regression was used to assess the effect of agents, introduced as fixed effects, on ICDAS score reduction categorized as reduction versus no reduction where reduction was recorded if the score changed from score 2 to score 1 or from score 1 to score 0 and no reduction was recorded if the scores remained unchanged or increased. This was done with and without controlling the confounders (age, sex, brushing with fluoridated toothpaste, dental visits, and sugar consumption, and the number of teeth with white spot lesions in each group). Significance level was set at *P* = 0.05. All tests were two tailed. Data were analyzed using IBM SPSS Statistics for Macintosh, Version 28.0. Armonk, NY: IBM Corp.

## Results

The study sample included 147 lesions in 66 patients. The mean (± SD) age was 13.46 (± 4.31) with 66.7% females. There were no significant differences among the groups except in the daily frequency of sugar intake and frequency of dental visits last year (*P* = 0.031 and 0.004, respectively, Table 2).

**Table 2:** Demographic description and oral health practices of study participants per group

		P11-4 (*N* = 22 patients, *n* = 44 lesions)	NSF (*N* = 22 patients, *n* = 44 lesions)	NaF (*N* = 22 patients, *n* = 59 lesions)	*P* value
Age: Mean (SD)		12.55 (3.54)	11.73 (2.87)	13.77 (4.6)	0.196
Sex n (%)	Male	9 (40.9%)	7 (31.8%)	8 (36.4%)	0.822
	Female	13 (59.1%)	15 (68.2%)	14 (63.6%)	
Frequency of sugar intake: Mean (SD)		1.5 (1.01)	2.45 (1.34)	2.09 (1.19)	0.031*
Last dental visit in the last year n (%)	Never	2 (9.1%)	4 (18.2%)	3 (13.6%)	0.004*
	Once	7 (31.8%)	8 (36.4%)	8 (36.4%)	
	Twice	6 (27.3%)	6 (27.3%)	9 (40.9%)	
	3 times	6 (27.3%)	4 (18.2%)	1 (4.5%)	
	4 times	1 (4.5%)	0	1 (4.5%)	
Frequency of brushing with fluoridated toothpaste n (%)	Never	2 (9.1%)	5 (22.7%)	4 (18.2%)	0.196
	More than 1 time per month	4 (18.2%)	3 (13.6%)	7 (31.8%)	
	Once or twice a week	8 (36.4%)	10 (45.5%)	5 (22.7%)	
	Once per day	6 (27.3%)	2 (9.1%)	3 (13.6%)	
	Twice per day	2 (9.1%)	2 (9.1%)	3 (13.6%)	
Number of teeth with white spot lesions: Mean (SD)		2 (1.35)	2 (1.45)	2.68 (1.36)	0.087

^*^Statistically significant *p* value < 0.05

There were significant differences in change of ICDAS scores among the three groups after 3 and 6 months (*p* = 0.005, Table 3). The reduction in ICDAS score increased in all groups in all periods with the greatest being after 12 months in the P11-4 group (54.5%), followed by NSF (47.7%) and the least in the NaF group (30.5%). There was no increase in ICDAS scores in the P11-4 and NSF groups and only 3.4% of lesion had increase in ICDAS scores in the NaF group after 12 months (Table 3).

**Table 3:** Change in ICDAS scores among the groups at different time intervals

		P11-4 (*N* = 22 patients, n = 44 lesions)	NSF (*N* = 22 patients, n = 44 lesions)	NaF (*N* = 22 patients, n = 59 lesions)	*Chi square (P* value)
		N (%)			
1 Month	Reduction	8 (18.2%)	5 (11.4%)	4 (6.8%)	4,603 (0.33)
	No change	36 (81.8%)	39 (88.6%)	54 (91.5%)	
	Increase	0	0	1 (1.7%)	
3 Months	Reduction	18 (40.9%)	14 (31.8%)	6 (10.2%)	14.703 (0.005)*
	No change	26 (59.1%)	30 (68.2%)	52 (88.1%)	
	Increase	0	0	1 (1.7%)	
6 Months	Reduction	24 (45.5%)	18 (40.9%)	9 (15.3)%	14.999 (0.005)*
	No change	20 (54.5%)	26 (59.1%)	48 (81.4%)	
	Increase	0	0	2 (3.4%)	
12 Months	Reduction	20 (54.5%)	21 (47.7%)	18 (30.5%)	8.793 (0.066)
	No change	24 (45.5%)	23 (52.3%)	39 (66.1%)	
	Increase	0	0	2 (3.4%)	

^*^Statistically significant *p* value < 0.05

Between group comparisons of lesion activity showed significant differences after 1 (*p* < 0.001), 3, 6 and 12 months (*p* = 0.009) (Table 4). All groups showed significant reduction in the percentages of active lesions using NYVAD LAA scores across time (*p* < 0.001). The reduction in lesion activity was observed in all groups till 3 months then became stable after that, with the greatest percentage of inactive lesions in the P11-4 group (100%), followed by NSF and NaF (81.4%) (Table 4).

**Table 4:** NYVAD scores between the groups at different time intervals

		P11-4 (*N* = 22 patients, n = 44 lesions)	NSF (*N* = 22 patients, n = 44 lesions)	NaF (*N* = 22 patients, n = 59 lesions)	*Chi square (P* value)
		N (%)			
Baseline	Inactive	21 (47.7%)	19 (43.2%)	22 (37.3%)	1.152 (0.562)
	Active	23 (52.3%)	25 (56.8%)	37 (62.7%)	
1 Month	Inactive	43 (97.7%)	33 (75%)	40 (67.8%)	14.145 (< 0.001)*
	Active	1 (%)	11 (25%)	19 (32.2%)	
3 Months	Inactive	44 (100%)	36 (81.8%)	48 (81.4%)	9.326 (0.009)*
	Active	0 (0%)	8 (16.2%)	11 (18.6%)	
6 Months	Inactive	44 (100%)	36 (81.8%)	48 (81.4%)	9.326 (0.009)*
	Active	0 (0%)	8 (16.2%)	11 (18.6%)	
12 Months	Inactive	44 (100%)	36 (81.8%)	48 (81.4%)	9.326 (0.009)*
	Active	0 (0%)	8 (16.2%)	11 (18.6%)	
*P* value		< 0.001*	< 0.001*	< 0.001*	

^*^Statistically significant *p* value < 0.05

**Fig. 2:**
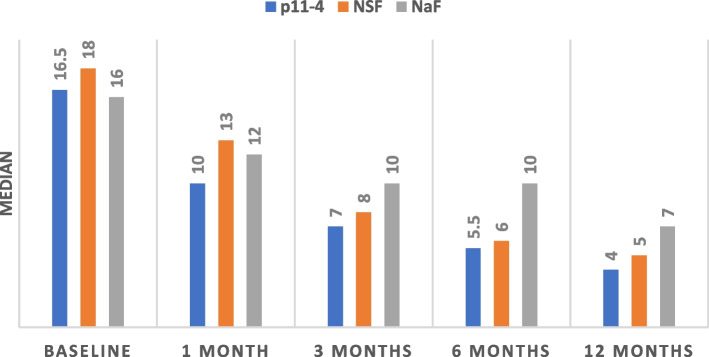
Diagnodent readings in the three groups at different follow up periods

There were significant differences in Diagnodent readings among the three groups in each follow up period (*p* < 0.001) and the reduction in median Diagnodent reading in each group across time was also significant (*p* = 0.001) with the lowest Diagnodent median reading observed in the P11-4 group after 12 months, Fig. 2.

The unadjusted multilevel binary logistic regression model showed that P11-4 (UOR = 2.34, 95% CI: 1.05, 5.22) and NSF (UOR = 1.78, 95% CI: 0.80, 3.97) had higher odds of inducing reduction in ICDAS scores than NaF varnish, although the difference was only statistically significant among the P11-4 group (*P* = 0.04). The adjusted multilevel binary logistic regression model showed that P11-4 (AOR = 2.26, 95% CI: 0.58, 8.77) and NSF (AOR = 2.12, 95% CI: 0.59, 7.64) had higher odds of inducing reduction in ICDAS scores than NaF varnish, although these differences were not statistically significant (*P* = 0.24 and 0.25, Table 5).

**Table 5:** Multilevel binary logistic regression assessing the effect of P11-4 and NSF compared to NaF on reduction versus no reduction in ICDAS score after 12 months

	**UOR**	**95% CI**	***P* value**	**AOR**	**95% CI**	***P* value**
**P11-4 vs NaF**	2.34	(1.05- 5.22)	0.04*	2.26	(0.58, 8.77)	0.24
**NSF vs NaF**	1.78	(0.80- 3.97)	0.16	2.12	(0.59, 7.64)	0.25
% Correctly classified	58.5%			82.3%		
Model *P* value	0.10			0.06		

Models are adjusted for age, sex, sugar frequency, number of white spot lesions, frequency of toothbrushing with fluoridated toothpaste and last dental visit

^*^Statistically significant *p* value < 0.05

## Discussion

This study showed that after one application of P11-4, there were no active lesions after 3 months with significantly lower Diagnodent readings than NaF at all follow-up periods. Also, the number of active lesions decreased steadily after one application of NSF and stabilized after 3 months with Diagnodent readings lower than NaF. However, the adjusted analysis showed that the reduction in ICDAS scores in the P11-4 and NSF groups was not significantly different from the NaF group. Thus, there is no support for rejecting the null hypothesis. Our study fills a knowledge gap by providing evidence on whether there are differences between P11-4 and NSF compared to NaF in remineralizing WSLs in permanent teeth. The findings may support the use of two applications of NaF, one application of NSF or P11-4 to control WSLs in permanent teeth depending on availability of agents and patient compliance although longer follow up periods may shed further light on the sustainability of the effects of the three agents.

In the present study, a longer follow up period was used than in most previous studies assessing the remineralization effect of the studied agents and an adjusted analysis was used that also accounted for clustering of teeth within persons. Because of these features, direct comparison with previous studies is difficult. For example, in the present study, there were no differences between the percentage of lesions with reduction of ICDAS scores after P11-4 or NSF compared to NaF after 12 months. This disagrees with Ali et al. [7] who reported significant difference between NSF and NaF in the number of WSLs in permanent teeth of young adults after 3 and 6 months. It also disagrees with Gözetici et.al [18], who showed that NaF induced greater regression of white spot buccal lesions than P11-4 after 6 months and Kamh et al. [17] who reported greater reduction in ICDAS II scores of WSLs in adults after 3 months when NaF and xylitol paste were used than when P11-4 was used. These differences highlight the need for studies with adequate duration and analysis techniques that take into consideration the differences that may exist among groups to draw valid conclusions about differences in the remineralization potential of caries preventive agents.

The present study showed that there were significantly fewer active lesions in the P11-4 and NSF groups than the NaF. This agrees with Ali et al. [7] who reported that the NSF had significant reduction in lesion activity up to 6 months. Moreover, Alkilzy et al. [12] and Doberdoli et al. [14] who showed that P11-4 resulted in greater reduction in lesion activity of early occlusal lesions in children and adolescents than NaF after 6 months. Most recently Keeper JH et al. [16] in their systematic review and meta-analysis has shown single application of P11-4 resulted in decreased lesion activity after 24 months. Our study fill a knowledge gap by proving evidence of the effectiveness of NSF in reducing lesion activity up to 12 months.

In the present study, there were significantly lower median Diagnodent readings in the NSF than the NaF group. This agrees with the only published clinical trial [6] comparing NSF and NaF varnishes in primary teeth that showed significant reduction of diagnodent readings in NSF group than NaF varnish after 3 months. The differences in the present study in Diagnodent readings between P11-4 and NaF also agree with systematic reviews [15, 16] concluding that up to 12 months, P11-4 showed significantly greater improvement in Diagnodent readings than NaF. The observed differences in the present study in Diagnodent readings among the groups when there were no significant differences in the percentage of lesions with reduction in ICDAS scores highlight the sensitivity of Diagnodent than ICDAS scoring in detecting changes in lesions limited to enamel [30].

Future clinical trials are needed to support the present findings. Further trials are also needed to examine the effects of the studied agents on occlusal and proximal surfaces which are common sites for dental caries in primary and permanent teeth. Studies with longer follow-up periods are also needed.

## Conclusion

The findings of this study highlight the remineralizing effect of P11-4 and NSF. After one year, there were no significant differences in the reduction of ICDAS scores among groups in adjusted analysis although P11-4 and NSF showed less caries activity and lower Diagnodent scores than the NaF group. Further studies are needed to guide clinical decision making to select appropriate remineralizing agents for WSLs in smooth surfaces.

## Data Availability

The datasets used and/or analysed during the current study are available from the corresponding author on reasonable request.

## References

[1] Lamont RJ, Egland PG, Tang Yi-Wei, Sussman M, Liu D, Poxton I, Schwartzman J (2015). Dental Caries. Molecular Medical Microbiology: Second Edition.

[2] Fisher J, Johnston S, Hewson N, Van Dijk W, Reich E, Eiselé JL, Bourgeois D (2012). FDI Global Caries Initiative; implementing a paradigm shift in dental practice and the global policy context. Int Dent J.

[3] Schmidlin P, Zobrist K, Attin T, Wegehaupt F (2016). In vitro re-hardening of artificial enamel caries lesions using enamel matrix proteins or self-assembling peptides. J Appl Oral Sci.

[4] García CR, Figueroa AL, Rubalcava CM (2011). Perspectives for the use of silver nanoparticles in dental practice. Int Dent J.

[5] Fung MHT, Duangthip D, Wong MCM, Lo ECM, Chu CH (2016). Arresting dentine caries with different concentration and periodicity of silver diamine fluoride. JDR Clin Transl Res.

[6] Giron CBT, Mariel-Cardenas J, Pierdant-Perez M, Hernandez-Sierra, Morales-Sanchez JE, Ruiz F. Effectiveness of a combined silver nanoparticles/fluoride varnish in dental remineralization in children: in vivo study. Superficies y Vacio. 2017;30:1–24.

[7] Ali A, Ismail H, Amin K (2022). Effect of nanosilver mouthwash on prevention of white spot lesions in patients undergoing fixed orthodontic treatment-a randomized double-blind clinical trial. J Dental Sci.

[8] Aggeli A, Bell M, Carrick LM, Fishwick CW, Harding R, Mawer P (2003). pH as a trigger of peptide ẞ-sheet self-assembly and reversible switching between nematic and isotropic phases. J Am Chem Soc.

[9] Aggeli A, Nyrkova IA, Bell M, Harding R, Carrick L, McLeish TC (2001). Hierarchical self-assembly of chiral rod-like molecules as a model for peptide ẞ-sheet tapes, ribbons, fibrils, and fibers. Proc Natl Acad Sci.

[10] Carrick LM, Aggeli A, Boden N, Fisher J, Ingham E, Waigh TA (2007). Effect of ionic strength on the self-assembly, morphology and gelation of pH responsive B- sheet tape-forming peptides. Tetrahedron.

[11] Brunton PA, Davies RPW, Burke JL, Smith A, Aggeli A, Brookes SJ (2013). Treatment of early caries lesions using biomimetic self-assembling peptides-a clinical safety trial. Br Dent J.

[12] Alkilzy M, Tarabaih A, Santamaria RM, Splieth CH (2018). Self-assembling peptide P11–4 and fluoride for regenerating enamel. J Dent Res.

[13] Bröseler F, Tietmann C, Bommer C, Drechsel T, Heinzel-Gutenbrunner M, & Jepsen S. Randomised clinical trial investigating self-assembling peptide P 11–4 in the treatment of early caries. Clin Oral Investig. 2019;1–10. 10.1007/s00784-019-02901-4 31037343

[14] Doberdoli D, Bommer C, Begzati A, Haliti F, Heinzel-Gutenbrunner M, Juric H (2020). Randomized clinical trial investigating self-assembling peptide P11–4 for treatment of early occlusal caries. Sci Rep.

[15] Wierichs RJ, Carvalho TS, Wolf TG (2021). Efficacy of a self-assembling peptide to remineralize initial caries lesions-A systematic review and meta-analysis. J Dent.

[16] Keeper JH, Skaret LJ, Thakkar-Samtani M, Heaton LJ, Sutherland C, Vela K, Amaechi BT, Jablonski-Momeni A, Young DA, MacLean JK, Weyant RJ. Systematic review and meta-analysis on the effect of self-assembling peptide P11–4 on initial caries lesions. medRxiv. 2022. 10.1016/j.adaj.2023.03.014 37245138

[17] Kamh RA, Niazy MA, El-Yasaky MA (2018). Clinical Performance and Remineralization Potential of Different Biomimitic Materials on White Spot Lesions. Al-Azhar Dental J for Girls.

[18] Gözetici B, Öztürk-Bozkurt F, Toz-Akalın T (2019). Comparative Evaluation of Resin Infiltration and Remineralisation of Noncavitated Smooth Surface Caries Lesions: 6-month Results. Oral Health Prev Dent.

[19] WHO. Child and adolescent health and development. 2019. Available from: http://www.searo.who.int/entity/child_adolescent/topics/adolescent_health/en/.

[20] Schlee M, Schad T, Koch JH, Cattin PC, Rathe F (2018). Clinical performance of self-assembling peptide P11–4 in the treatment of initial proximal carious lesions: A practice-based case series. J Investig Clin Dent.

[21] Muñoz-Millán P, Zaror C, Espinoza-Espinoza G, Vergara-Gonzalez C, Muñoz S, Atala-Acevedo C (2018). Effectiveness of fluoride varnish in preventing early childhood caries in rural areas without access to fluoridated drinking water: A randomized control trial. Commun Dent Oral Epidemiol.

[22] Saghaei M (2004). Random allocation software for parallel group randomized trials. BMC Med Res Methodol.

[23] Curodont^TM^. Biomedical products for tooth preservation. 2019. Available from: https://www.straumann.com/en/landing/curodont.html.

[24] Wei D, Sun W, Qian W, Ye Y, Ma X (2009). The synthesis of chitosan-based silver nanoparticles and their antibacterial activity. Carbohyd Res.

[25] Dos Santos Jr VE, Vasconcelos Filho A, Targino AGR, Flores MAP, Galembeck A, Caldas AF (2014). A new “Silver-Bullet” to treat caries in children-Nano Silver Fluoride: a randomised clinical trial. J Dent.

[26] Cerón-Bastidas XA (2015). The ICDAS system as a complementary method for the diagnosis of dental caries. CES Odontol.

[27] Braga MM, Benedetto MSD, Imparato JC, Mendes FM (2010). New methodology to assess activity status of occlusal caries in primary teeth using laser fluorescence device. J Biomed Opt.

[28] Shinohara T, Takase Y, Amagai T, Haruyama C, Igarashi A, Kukidome N (2006). Criteria for a diagnosis of caries through the DIAGNOdent. Photomed Laser Surg.

[29] World Health Organization. Oral health surveys: basic methods. World Health Organization. 2013. Available at: https://www.who.int/publications/i/item/9789241548649.

[30] Minuesa-García E, Iranzo-Cortés JE, Almerich-Torres T, Bellot-Arcís C, Montiel-Company JM, Almerich-Silla JM (2022). Diagnostic Validity in Occlusal Caries Detection of ICDAS II, DIAGNOdent, Radiography and a Combination of the Three Methods: An In Vitro Study. J Clin Med.

